# Acetylcorynoline Impairs the Maturation of Mouse Bone Marrow-Derived Dendritic Cells via Suppression of IκB Kinase and Mitogen-Activated Protein Kinase Activities

**DOI:** 10.1371/journal.pone.0058398

**Published:** 2013-03-05

**Authors:** Ru-Huei Fu, Yu-Chi Wang, Shih-Ping Liu, Ching-Liang Chu, Rong-Tzong Tsai, Yu-Chen Ho, Wen-Lin Chang, Shao-Chih Chiu, Horng-Jyh Harn, Woei-Cherng Shyu, Shinn-Zong Lin

**Affiliations:** 1 Graduate Institute of Immunology, China Medical University, Taichung, Taiwan; 2 Center for Neuropsychiatry, China Medical University Hospital, Taichung, Taiwan; 3 Biomedical Technology and Device Research Laboratories, Industrial Technology Research Institute, Hsinchu, Taiwan; 4 Graduate Institute of Basic Medical Science, China Medical University, Taichung, Taiwan; 5 Graduate Institute of Immunology, National Taiwan University, Taipei, Taiwan; 6 Institute of Biochemistry and Biotechnology, Chung Shan Medical University, Taichung, Taiwan; 7 China Medical University Beigang Hospital, Yunlin, Taiwan; University of Cincinnati, United States of America

## Abstract

**Background:**

Dendritic cells (DCs) are major modulators in the immune system. One active field of research is the manipulation of DCs as pharmacological targets to screen novel biological modifiers for the treatment of inflammatory and autoimmune disorders. Acetylcorynoline is the major alkaloid component derived from *Corydalis bungeana* herbs. We assessed the capability of acetylcorynoline to regulate lipopolysaccharide (LPS)-stimulated activation of mouse bone marrow-derived DCs.

**Methodology/Principal Findings:**

Our experimental data showed that treatment with up to 20 µM acetylcorynoline does not cause cytotoxicity in cells. Acetylcorynoline significantly inhibited the secretion of tumor necrosis factor-α, interleukin-6, and interleukin-12p70 by LPS-stimulated DCs. The expression of LPS-induced major histocompatibility complex class II, CD40, and CD86 on DCs was also decreased by acetylcorynoline, and the endocytic capacity of LPS-stimulated DCs was restored by acetylcorynoline. In addition, LPS-stimulated DC-elicited allogeneic T-cell proliferation was blocked by acetylcorynoline, and the migratory ability of LPS-stimulated DCs was reduced by acetylcorynoline. Moreover, acetylcorynoline significantly inhibits LPS-induced activation of IκB kinase and mitogen-activated protein kinase. Importantly, administration of acetylcorynoline significantly attenuates 2,4-dinitro-1-fluorobenzene-induced delayed-type hypersensitivity.

**Conclusions/Significance:**

Acetylcorynoline may be one of the potent immunosuppressive agents through the blockage of DC maturation and function.

## Introduction

**Figure 1:**
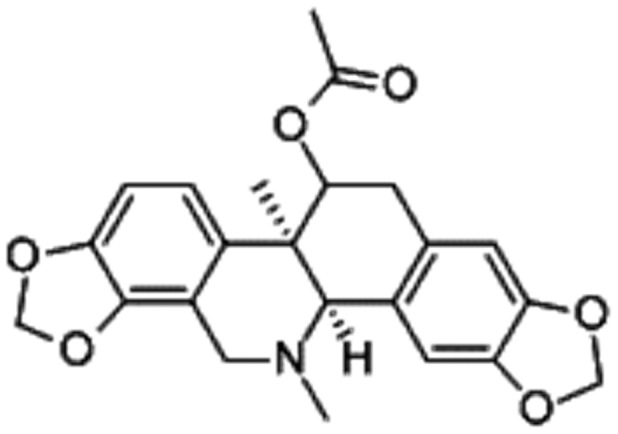
Chemical structure of acetylcorynoline.

*Corydalis bungeana* Turcz. (Papaveraceae) is a perennial herb scattered over the region of Northeast Asia [1]. The dried whole plant is referred to as *C. Bungeanae* Herba in traditional Chinese medicine and is officially recorded in the *Chinese Pharmacopoeia* [2]. It has been listed for treatments such as upper respiratory tract infections, tonsillitis, influenza, bronchitis, pyelonephritis, and acute nephritis [1]. Acetylcorynoline (Figure 1) is the major alkaloid component derived from *C. bungeana* herbs. *In vitro* studies showed that acetylcorynoline reduced carbon tetrachloride (CCl_4_)-induced microsomal lipid peroxidation and CCl_4_ conversion to carbon monoxide in liver microsomes. Oral administration of acetylcorynoline has been shown to significantly decrease elevated serum levels of glutamate pyruvate transaminase and liver damage induced by injection of CCl_4_, acetaminophen, or thioacetamide in mice [3].

Dendritic cells (DCs) are the main professional antigen-presenting cells, functioning as messengers for communication between innate and adaptive immunity [4]. For that reason, DCs have been used in treating infectious diseases and malignant tumors. In addition, DCs play a major role in immune control, including tolerance induction and prevention of autoimmunity [5]. DCs have two functional stages. Immature DCs are differentiated from bone marrow progenitor cells and exist in blood or tissues in contact with the outside environment. These cells display high endocytic capability and low T-cell stimulation potential. After antigen uptake, DCs process selected foreign peptides, transfer them to the surface, and turn into mature DCs. They then travel to the secondary lymph organs where they activate T cells. In the innate immune response, DCs can use pathogen-associated molecular patterns—ordinary constituents of microbes—to recognize pathogenic components. One pathogen-associated molecular pattern is lipopolysaccharide (LPS), which is derived from the outer membrane of gram-negative bacteria and has been considered a major cause of septic shock [6]. LPS combines with LPS-binding protein and CD14 and then specially binds to Toll-like receptor 4 (TLR4) on DCs. After activation of TLR4, DCs initiate the process of maturation, including production of proinflammatory cytokines (e.g., tumor necrosis factor [TNF]-α, interleukin [IL]-6, and IL-12), up-regulation of surface effector molecules (e.g., major histocompatibility complex [MHC] class II, CD40, CD80, and CD86), loss of endocytotic/phagocytic capability, and gain of competence to transfer into secondary lymphoid organs by C-C chemokine receptor type 7 (CCR7) expression, which strengthens their antigen-presenting function and triggers specific T-cell immune responses [7].

Several intracellular signaling pathways are involved in the engagement of TLR4 activities, including the IκB kinase (IKK)/nuclear factor-κB (NF-κB) pathway [8], [9], the phosphatidylinositol 3-kinase (PI3K)/Akt pathway [10], and three mitogen-activated protein kinase (MAPK) pathways [11], [12]: c-Jun N-terminal kinase (JNK), extracellular signal-regulated kinase (ERK), and p38, which direct the expression of various genes related to DC maturation.

Because DCs are important immunomodulators, regulating their activity may be a valuable approach for treating inflammatory and autoimmune disorders [13]. Thus, an active field of study is the handling of DCs as pharmacological targets to search new biological modifiers of immune responses [14], [15], [16], [17], [18], [19], [20], [21], [22], [23].

To date, no research has examined the immunomodulatory properties of acetylcorynoline in DCs. In this study, we hypothesized that acetylcorynoline can affect DC maturation. To investigate this hypothesis, we explored the influences of acetylcorynoline on the maturation of DC and the related signal pathway by using a model of LPS-stimulated mouse bone marrow-derived DCs (mBM-DCs).

## Results

### Effect of acetylcorynoline on cell viability

**Figure 2:**
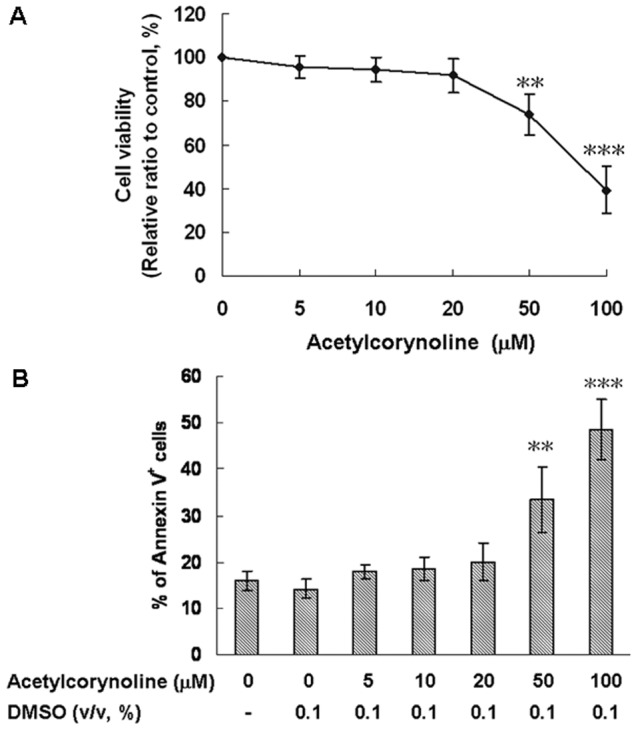
Effects of acetylcorynoline on the viability of mBM-DCs. mBM-DCs were treated with serially diluted acetylcorynoline for 24 h. The viability of cells was determined by propidium iodide staining/flow cytometry assay (A). The apoptosis of cells was determined by annexin V-fluorescein staining/flow cytometry assay (B), as described in the text. The data represent the mean ± SD (n = 3). An asterisk (*) indicates significant differences between acetylcorynoline untreated control samples and acetylcorynoline treated samples (^**^ *p*<0.01, ^***^ *p*<0.001).

In the current study, the immunomodulatory effects of acetylcorynoline were analyzed by using mBM-DCs. A propidium iodide staining/flow cytometry assay and an annexin V-fluorescein staining/flow cytometry assay were used to evaluate the cytotoxicity of acetylcorynoline. Cell viability and apoptosis were not significantly changed by 24-h treatment with up to 20 µM acetylcorynoline (Figure 2A and B). In the experiments following this assay, cells were treated with acetylcorynoline at concentrations of up to 20 µM.

### Inhibitory effect of acetylcorynoline on LPS-induced TNF-α, IL-6, and IL-12p70 secretion

**Figure 3:**
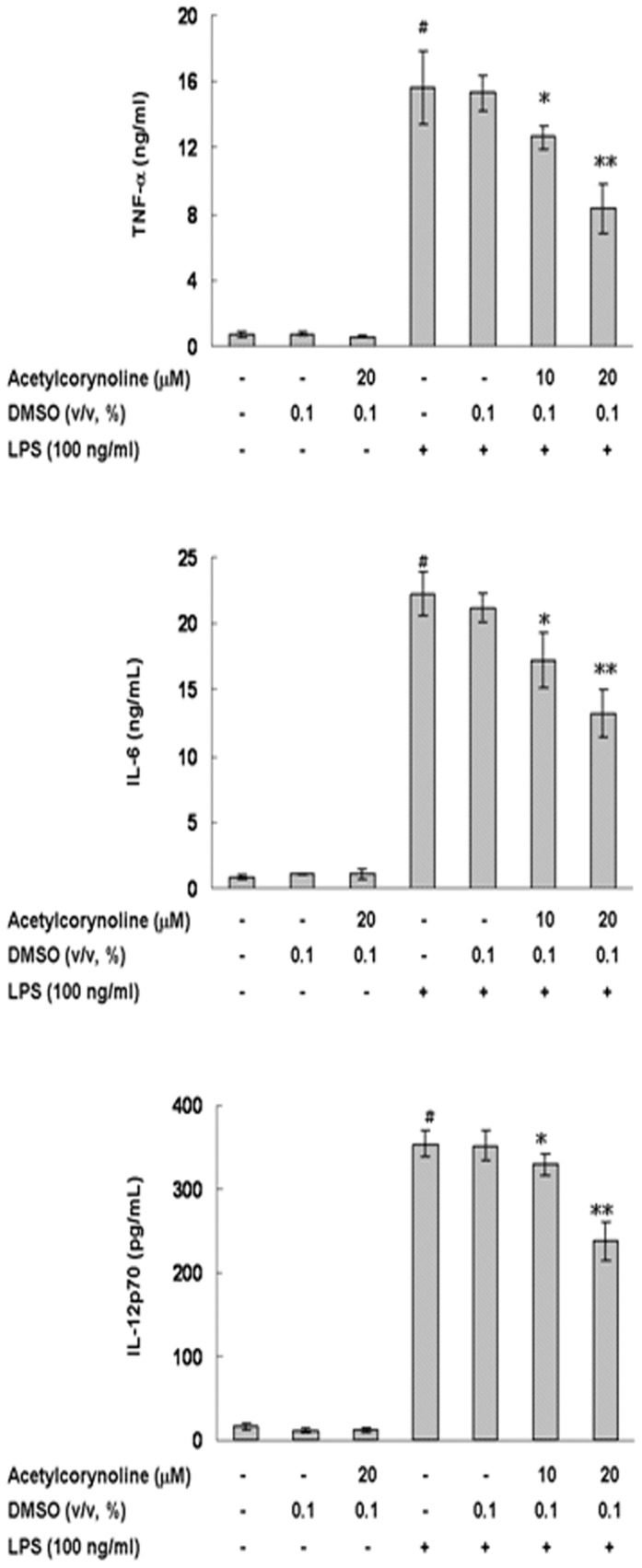
Inhibitory effects of acetylcorynoline on TNF-α, IL-6, and IL-12p70 secretion in LPS-stimulated mBM-DCs. mBM-DCs were pretreated with 10 or 20 µM acetylcorynoline. After 1 h of incubation, the cells were washed, followed by stimulation with 100 ng/ml LPS for 24 h (6 h for TNF-α). Media were collected and assayed for TNF-α, IL-6 and IL-12p70 levels by using as ELISA kit. The data represent the mean ± SD (n = 3). A hash (#) indicates significant differences between LPS-stimulated and unstimulated cells (*p*<0.001); an asterisk (*) indicates significant differences between the LPS-stimulated control samples and acetylcorynoline-pretreated, LPS-stimulated samples (^*^ *p*<0.05, ^**^ *p*<0.01).

During maturation and activation, DCs alter their phenotype and functional properties. TNF-α, IL-6, and IL-12 are three key proinflammatory cytokines that induce the expression of costimulatory/accessory molecules on DCs and strengthen DC-mediated T-cell responses [7]. Cytokine production was measured by ELISA. In unstimulated mBM-DCs, 20 µM acetylcorynoline did not change TNF-α, IL-6, and IL12p70 production. Treatment with LPS caused a 22-fold (*p*<0.001), 25-fold (*p*<0.001), and 22-fold increase (*p*<0.001) in the release of TNF-α, IL-6, and IL-12p70, respectively. Acetylcorynoline decreased the secretion of TNF-α, IL-6, and IL12p70 in a concentration-dependent manner. At 20 µM acetylcorynoline, LPS-stimulated TNF-α secretion decreased by about 47% (*p*<0.01), IL-6 secretion by about 40% (*p*<0.01), and IL-12p70 secretion by about 32% (*p*<0.01) (Figure 3).

### Inhibitory effect of acetylcorynoline on LPS-induced surface marker expression

**Figure 4:**
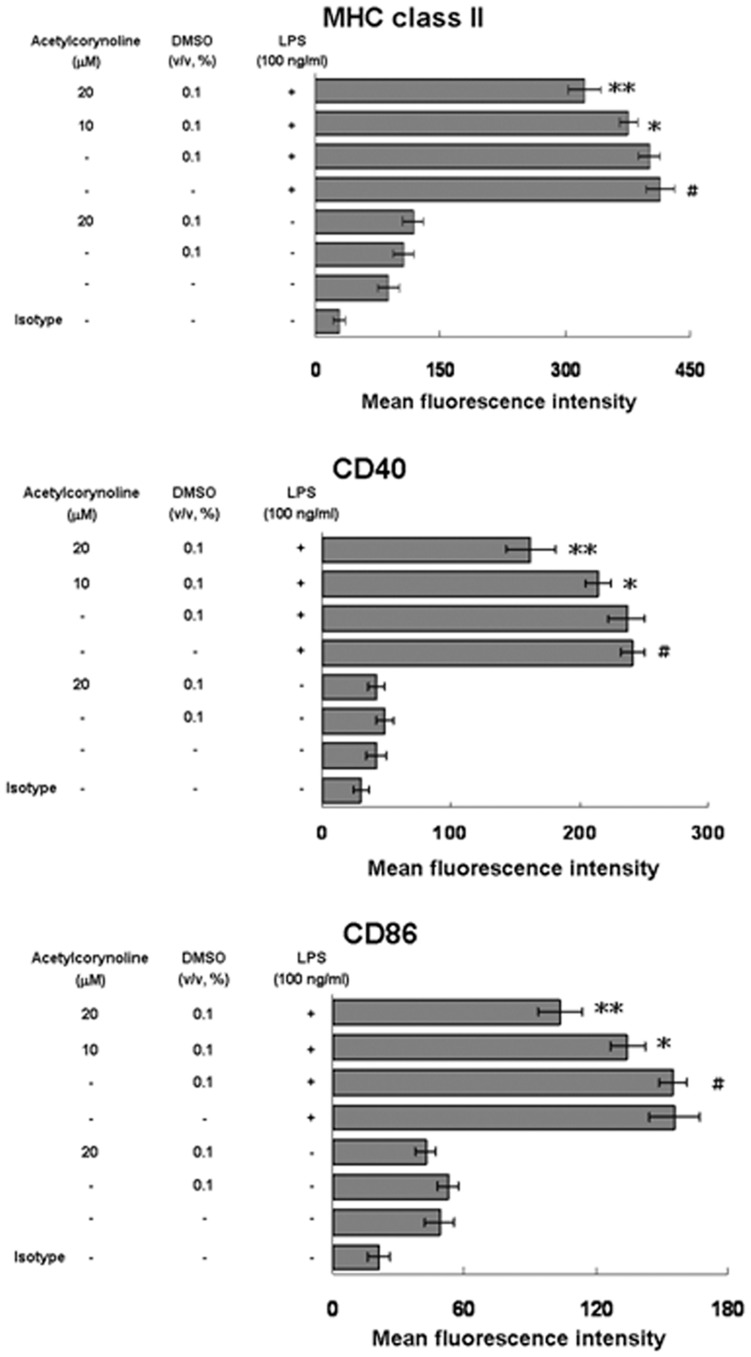
Inhibitory effects of acetylcorynoline on MHC class II, CD40, and CD86 expression in LPS-stimulated mBM-DCs. mBM-DCs were pretreated with 10 or 20 µM acetylcorynoline. After 1 h of incubation, the cells were washed, followed by stimulation with 100 ng/ml LPS for 16 h. The expression of MHC class II, CD40 and CD86 on CD11c^+^ cells was determined by flow cytometry. The data are represented as the mean fluorescent intensity ± SD (n = 3). A hash (#) indicates significant differences between LPS-stimulated and unstimulated cells (*p*<0.001); an asterisk (*) indicates significant differences between the LPS-stimulated control samples and acetylcorynoline-pretreated, LPS-stimulated samples (^*^ *p*<0.05, ^**^ *p*<0.01).

Interactions between surface effector molecules on DCs and their ligands are essential for the full activation of T cells. MHC class II presents the extracellular antigen peptide to a CD4^+^ T cell. The CD40 binds CD 154 on the CD4^+^ T cell to gain an activation signal, increasing antigen presentation and the expression of other costimulatory molecules. CD86 binds CD28 on the helper T cell for T-cell priming and survival [7]. The effect of acetylcorynoline on surface-specific molecules of activated DCs was analyzed by flow cytometry. The level of MHC class II, CD40, and CD86 was estimated by fluorescence intensity. Unstimulated mBM-DCs treated with acetylcorynoline did not change MHC class II, CD40, and CD86 expression. However, LPS-stimulated mBM-DCs greatly increased the expression of MHC class II, CD40, and CD86 (*p*<0.001). Acetylcorynoline treatment of 20 µM blocked MHC class II (*p*<0.01), CD40 (*p*<0.01), and CD86 (*p*<0.01) expression on mBM-DCs stimulated with LPS (Figure 4).

### Inhibitory effect of acetylcorynoline on endocytic capacity

**Figure 5:**
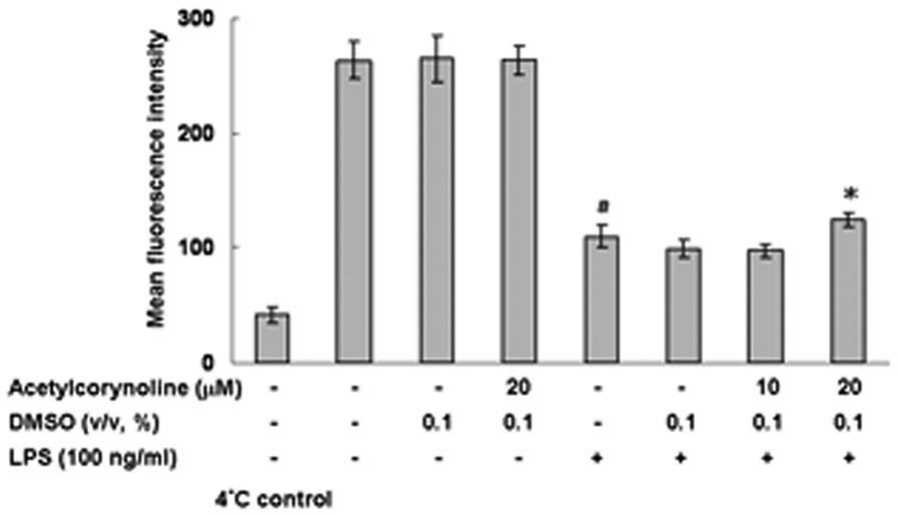
Inhibitory effects of acetylcorynoline on antigen uptake in LPS-stimulated mBM-DCs. mBM-DCs were pretreated with 10 or 20 µM acetylcorynoline. After 1 h of incubation, the cells were washed, followed by stimulation with 100 ng/ml LPS for 16 h. Cell were then incubated with 1 mg/ml FITC-dextran at 37°C for 1 h and analysed by flow cytometry. Control experiments were performed at 4°C for 1 h. The data are represented as the mean fluorescent intensity the mean ± SD (n = 3). A hash (#) indicates significant differences between LPS-stimulated and unstimulated cells (*p*<0.001); an asterisk (*) indicates significant differences between the LPS-stimulated control samples and acetylcorynoline-pretreated, LPS-stimulated samples (^*^ *p*<0.05).

The competence for antigen uptake in immature DCs is efficient but lost on maturation [7]. Therefore, the inhibition of DC maturation can be assessed by endocytosis assay. We measured the antigen uptake of mBM-DCs by using FITC-dextran/flow cytometry. UP-regulated dextran uptake was observed in acetylcorynoline-pretreated, LPS-stimulated mBM-DCs (20 µM, *p*<0.05) (Figure 5). Acetylcorynoline partly recovered the endocytosis capability of LPS-stimulated mBM-DCs.

### Inhibitory effect of acetylcorynoline on allostimulatory capacity

**Figure 6:**
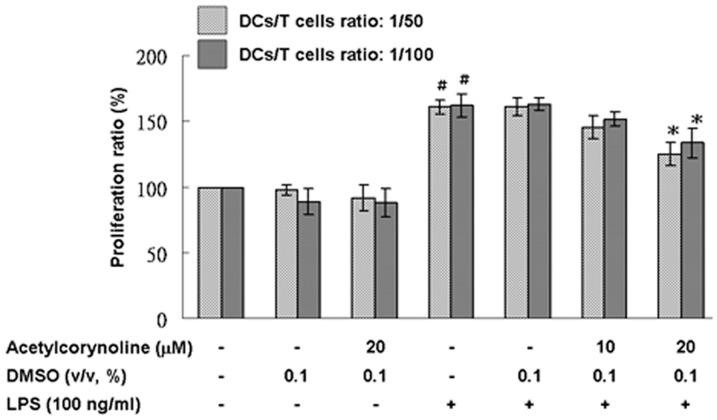
Inhibitory effects of acetylcorynoline on the proliferation of naïve allogeneic T lymphocytes by LPS-stimulated mBM-DCs. mBM-DCs were pretreated with 10 or 20 µM acetylcorynoline. After 1 h of incubation, the cells were washed, followed by stimulation with 100 ng/ml LPS for 16 h. DCs were then washed and incubated with 25 µg/ml mitomycin C for 30 min at 37°C. Finally, the cells were washed and diluted with the prepared splenocytes in a ratio of 1∶50 and 1∶100 in culture plates for 3 days. The proliferation of T cells was assessed by the MTT assay. DCs were sufficiently fixed with mitomycin C according to a DC-only control (data not shown). The values of unstimulated dendritic cells served as control values in the calculation of percentage of proliferation. The data represent the mean ± SD (n = 3). A hash (#) indicates significant differences between LPS-stimulated and unstimulated cells (*p*<0.001); an asterisk (*) indicates significant differences between the LPS-stimulated control samples and acetylcorynoline-pretreated, LPS-stimulated samples (^*^ *p*<0.05).

To evaluate the effect of acetylcorynoline on the allostimulatory capacity of mBM-DCs, we used a mixed lymphocyte reaction/MTT assay and splenocytes from BALB/c mice as responder T cells. As shown in Figure 6, acetylcorynoline-pretreated, LPS-stimulated mBM-DCs had low stimulatory capacity as compared with untreated mBM-DCs (20 µM, *p*<0.05), indicating that acetylcorynoline treatment arrested the allostimulatory capacity of stimulated mBM-DCs.

### Inhibitory effect of acetylcorynoline on migration activity

**Figure 7:**
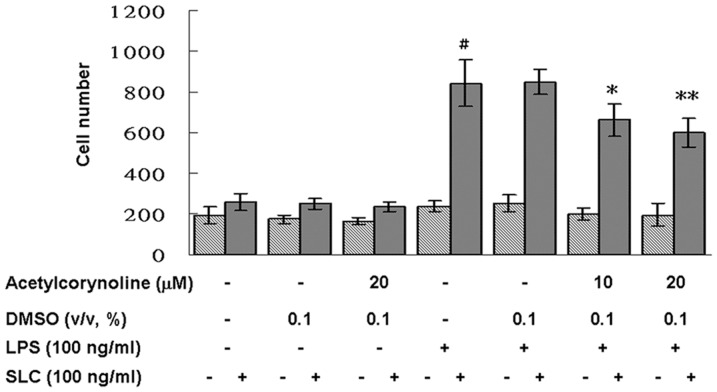
Inhibitory effects of acetylcorynoline on the migratory capacity of LPS-stimulated mBM-DCs. mBM-DCs were pretreated with 10 or 20 µM acetylcorynoline. After 1 h of incubation, the cells were washed, followed by stimulation with 100 ng/ml LPS for 16 h. The *in vitro* migration of DCs in response to 100 ng/ml SLC/CCL21 was determined by chemotaxix assay and flow cytometry. The data represent the mean ± SD (n = 3). A hash (#) indicates significant differences between LPS-stimulated and unstimulated cells (*p*<0.001); an asterisk (*) indicates significant differences between the LPS-stimulated control samples and acetylcorynoline-pretreated, LPS-stimulated samples (^*^ *p*<0.05, ^**^ *p*<0.01).

To examine whether acetylcorynoline affected the migration of DCs, we studied mBM-DCs *in vitro* for their migration in response to SLC/CCL21 in Transwell chambers. A comparison of the migration of acetylcorynoline-pretreated (20 µM) and nonpretreated mBM-DCs in response to SLC/CCL21 showed that acetylcorynoline decreased migration of LPS-stimulated DCs (*p*<0.01) (Figure 7). Therefore, both phenotypic and functional maturation of mBM-DCs was blocked by acetylcorynoline treatment.

### Inhibitory effect of acetylcorynoline on LPS-induced NF-κB p65 translocation

**Figure 8:**
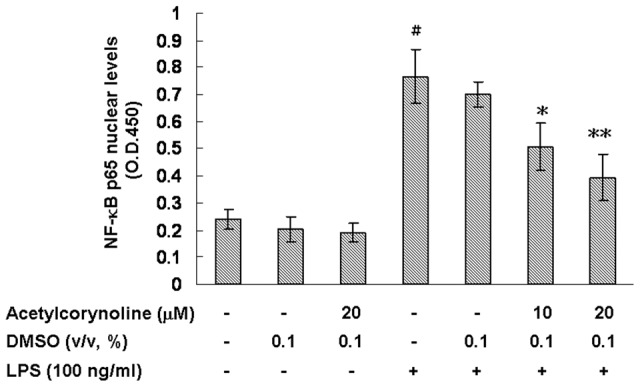
Inhibitory effect of acetylcorynoline on the translocation of NF-κB p65 in LPS-stimulated mBM-DCs. mBM-DCs were pretreated with 10 or 20 µM acetylcorynoline. After 1 h of incubation, the cells were washed, followed by stimulation with 100 ng/ml LPS for 1 h. Cells were lysed and the nuclear fraction determined for relative binding activity of NF-κB p65 by using the Universal EZ-TFA Transcription Factor Assay Colorimetric kit. The data represent the mean ± SD (n = 3). A hash (#) indicates significant differences between LPS-stimulated and unstimulated cells (*p*<0.001); an asterisk (*) indicates significant differences between the LPS-stimulated control samples and acetylcorynoline-pretreated, LPS-stimulated samples (^*^ *p*<0.05, ^**^ *p*<0.01).

Translocation of NF-κB from the cytosol to the nucleus is fundamental for LPS-induced activation of DCs [9], [24]. Given that acetylcorynoline attenuated the LPS-activation of DCs in our studies, we tested the effects of acetylcorynoline on NF-κB p65 levels in the nucleus. As shown in Figure 8, LPS-stimulated DCs elevated the NF-κB p65 levels in the nucleus (*p*<0.001), whereas acetylcorynoline treatment of 20 µM lowered nuclear NF-κB p65 levels in the LPS-stimulated DCs in a concentration-dependent manner (*p*<0.01).

### Inhibitory effect of acetylcorynoline on LPS-induced IKK and MAPK phosphorylation in the cytoplasm

**Figure 9:**
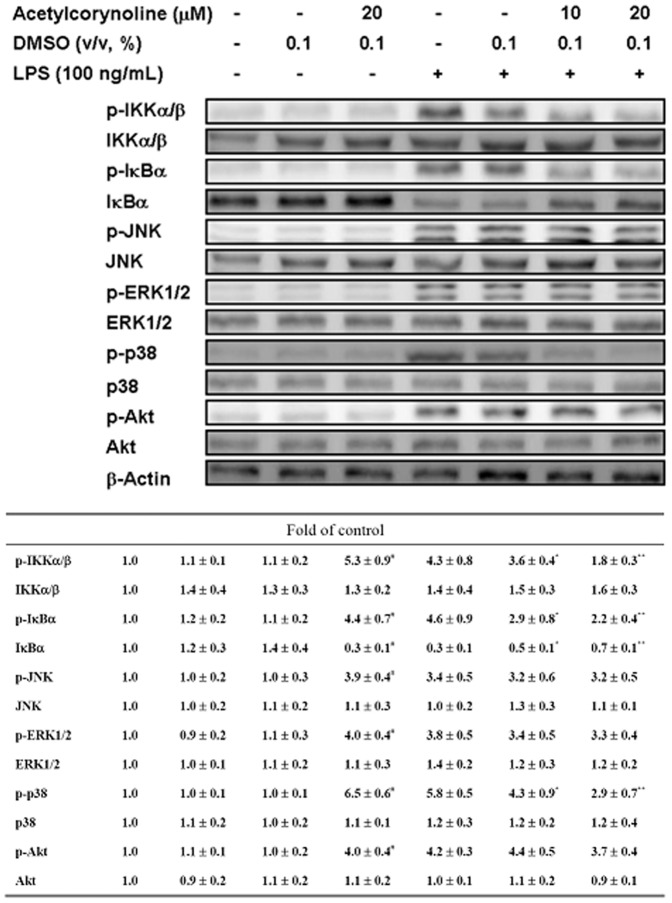
Inhibitory effect of acetylcorynoline on the activation of the IKK/NF-κB and MAPK signaling pathways in LPS-stimulated mBM-DCs. mBM-DCs were pretreated with 10 or 20 µM acetylcorynoline. After 1 h of incubation, the cells were washed, followed by stimulation with 100 ng/ml LPS for 1 h. Cells were lysed and phosphorylation levels of IKKα/β, IκBα, JNK, ERK1/2, p38, and Akt were analyzed by western blot analysis. The expression of β-actin was used as an internal control. One representative result from three independent experiments is shown. The relative fold in protein level was calculated as the level in pretreated cells relative to that in acetylcorynoline-untreated, LPS-unstimulated controls. The data represent the mean ± SD (n = 3). A hash (#) indicates significant differences between LPS-stimulated and unstimulated cells (*p*<0.001); an asterisk (*) indicates significant differences between the LPS-stimulated control samples and acetylcorynoline-pretreated, LPS-stimulated samples (^*^ *p*<0.05, ^**^ *p*<0.01).

**Figure 10:**
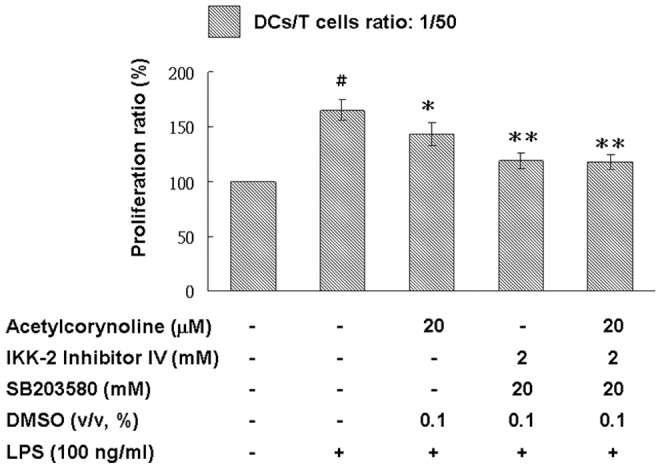
Effects of IKK and p38 MAPK pathways on allostimulatory ability of acetylcorynoline-pretreated, LPS-stimulated mBM-DCs. mBM-DCs were preincubated with the indicated inhibitors for 30 min, and then treated with or without 20 µM acetylcorynoline. After 1 h of incubation, the cells were washed, followed by stimulation with 100 ng/ml LPS for 16 h. DCs were then washed and incubated with 25 µg/ml mitomycin C for 30 min at 37°C. Finally, the cells were washed and diluted with the prepared splenocytes in a ratio of 1∶50 in culture plates for 3 days. The proliferation of T cells was assessed by the MTT assay. The values of unstimulated DCs served as control values in the calculation of percentage of proliferation. The data represent the mean ± SD (n = 3). A hash (#) indicates significant differences between LPS-stimulated and unstimulated cells (*p*<0.001); an asterisk (*) indicates significant differences between the LPS-stimulated control samples and inhibitors-preincubated (or inhibitors/acetylcorynoline-preincubated), LPS-stimulated samples (^*^ *p*<0.05, ^**^ *p*<0.01).

Several signaling pathways are involved in DC maturation [9]. To further examine the effect of acetylcorynoline in IKK/NF-κB, PI3K/Akt, and MAPK pathways, we studied the phosphorylation levels of major signaling factors involved in the activation of DC by using Western blot analysis. As shown in Figure 9, LPS obviously induced IKKα/β, IκBα, and p38 phosphorylation and promoted IκBα degradation in mBM-DCs, which is blocked in acetylcorynoline-pretreated cells in a concentration-dependent manner (20 µM, *p*<0.01). LPS-induced JNK, ERK1/2, and Akt phosphorylation was not arrested by acetylcorynoline treatment. Moreover, blocking the IKK and p38 MAPK signaling molecules with IKK-2 Inhibitor IV and SB20358, respectively, completely abolished the capacity of acetylcorynoline to inhibit DC-induced allogeneic T cell proliferation (Figure 10). Therefore, acetylcorynoline controlled the activation of NF-κB and MAPK signal transduction pathways that participated in DC maturation by blocking IKK and p38 MAPK activity.

### Inhibitory effect of acetylcorynoline on contact hypersensitivity (CHS) responses

**Figure 11:**
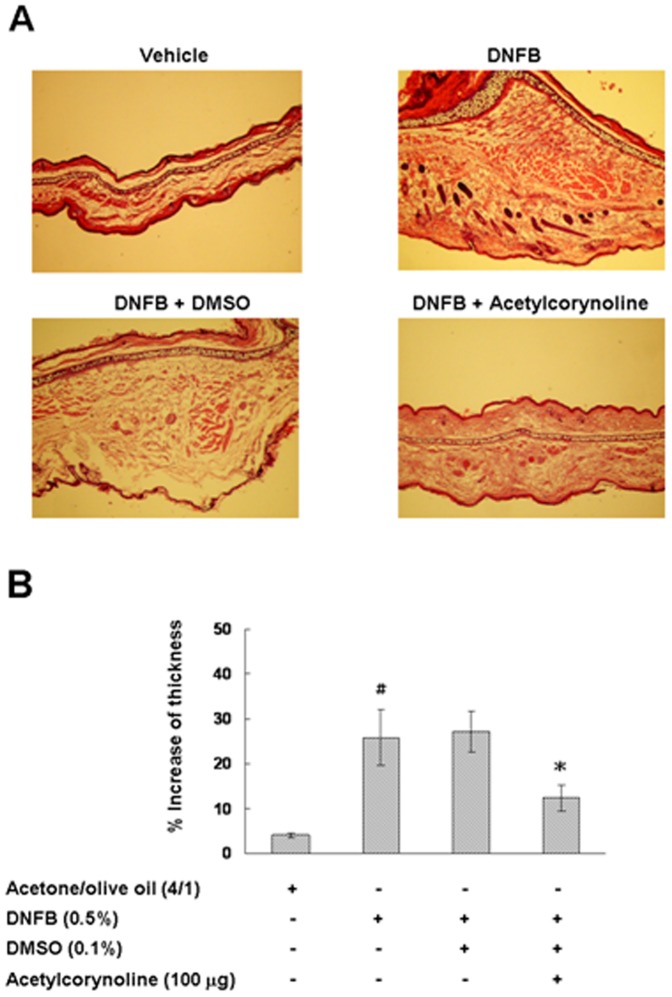
Inhibitory effect of acetylcorynoline on the contact hypersensitivity response in mice. Contact hypersensitivity response was showed by hematoxylin and eosin staining (A), and thickness of the challenged ear was calculated (original magnification times 40) (B). Mice that were not sensitized but were challenged with DNFB provided as negative controls. One representative result from three independent experiments is shown. The data represent the mean ± SD (n = 3). A hash (#) indicates significant differences between unsensitized and sensitized mice (*p*<0.001); an asterisk (*) indicates significant differences between the DNFB-challenged control samples and DNFB plus acetylcorynoline-challenged samples (^*^ *p*<0.05).

We confirmed an obstructive effect of acetylcorynoline on DC maturation, which suggests that acetylcorynoline may prevent DC-mediated disorders. Thus, we carried out 2.4-dinitro-1-fluorobenzene (DNFB)-induced CHS response as a model to examine this hypothesis. Mice were sensitized by painting DNFB in the absence or presence of acetylcorynoline directly onto the belly. The CHS response to DNFB was then tested. The ears were visibly swollen in DNFB-sensitized but not in DNFB plus acetylcorynoline-sensitized mice, whereas DMSO had no influence on DNFB-sensitized mice (Figure 11), implying that acetylcorynoline blocks the DC-mediated sensitization in CHS. These observations indicate that acetylcorynoline has the potential to prevent delayed-type hypersensitive disorders, much as allergic contact dermatitis.

## Discussion

Our investigational results demonstrate that acetylcorynoline reduces the secretion of LPS-induced preinflammatory cytokines TNF-α, IL-6, and IL-12p70 by DCs; attenuates LPS-induced expression of MHC class II, CD40, and CD86 molecules by DCs; and arrests LPS-induced migration of DCs and LPS-induced, DC-triggered allogeneic T-cell proliferation. To the best of our knowledge, this is the first report of the immunosuppressive function of acetylcorynoline on DC maturation. TNF-α plays a key role in control of the inflammatory response such as inducing secretion of IL-1, IL-6, transforming growth factor and platelet-derived growth factor and stimulating expression of adhesion molecules. Moreover, TNF-α also enhances the production of nitrogen species and reactive oxygen by leucocytes. [25]. IL-6 has been implicated in a wide range of biological functions, including proliferation and differentiation of lymphocytes, antibody production in activated B cells, modulation of Th1-associated cytokine expression and IL-2 receptor, upregulation of acute phase proteins in the liver [26]. Hence, acetylcorynoline blocked the expression of TNF-*α* and IL-6 to inhibit inflammatory response. Downregulation of CD40 molecules on DCs could damage antigen-presenting capability, lessen cytokine production and suppress CD80 and CD86 expression [27].

Endocytosis is a process in which extracellular antigens and immune complexes are internalized [28]. The reduced endocytic ability in DCs is maturation dependent. Our data showed that acetylcorynoline partly recovered antigen uptake competence. Endocytosis of immature DCs is through several mechanisms, including constitutive macropinocytosis (nonspecific uptake) and receptor-mediated endocytosis and phagocytosis (specific uptake) [29]. In this study, endocytosis assay involved in exposing cells with high concentration of endocytic tracers when measure macropinocytosis. Active form of the Rho GTPase, Cdc42 has been shown part regulating macropinocytosis [30]. Acetylcorynoline may up-regulate the active form of Cdc42 and promote endocytosis of mature DCs. The detail mechanism will require further examination. The migration of DCs is decreased by acetylcorynoline. CCR7 expression has been indicated to play a major role in controlling the DC migration [31]. However, acetylcorynoline had no observable effect on the expression of CCR7 in DCs after LPS stimulation (data not shown), suggesting that other mechanisms may contribute to the hindering effect of acetylcorynoline on DC migration, which will require further examination.

The cytoplasmic proteins, IκBs, regulate the nuclear translocation and activity of NF-κB. The IκBs bind with NF-κB dimers, so blocking their nuclear translocation. Triggering of TLR4 activity by the LPS complex promotes phosphorylation of IκBs, succeeded by their degradation and the release of NF-κB, which is consequently translocated into the nucleus. NF-κB controls the expression of proinflammatory mediators, including cytokines, costimulatory molecules, and adhesion molecules in DCs, and is upregulated when DCs mature [8], [9], [24]. In this study, we found that acetylcorynoline influences multiple intracellular signaling pathways downstream of TLR4 in DCs. Acetylcorynoline arrested the activation of NF-κB by blocking the degradation of IκBα and the nuclear translocation of p65 in LPS-stimulated DCs. Activation of NF-κB in DC has been revealed to up-regulate the DC costimulatory molecules, CD80, CD86, and HLA-DR as well as the maturation marker CD83 [32]. MAPK signaling pathways also play an important role in DC maturation. In this research, p38 activity was blocked by acetylcorynoline in LPS-stimulated DCs. p38 has been shown to induce the IL-12 [33] and CD83 [34] in DC. p38 also up-regulate the cellular inhibitor of apoptosis protein 2, cIAP2, thus indicating a role for maintaining DC survival [35]. Consequently, we have identified that acetylcorynoline impairs LPS-induced activation of DCs, at least in part, through suppression of IKK and p38 MAPK activity by inhibitor assay. Moreover, acetylcorynoline may compete with DC for LPS binding causing inhibition of LPS-stimulated DC maturation. The outcome of the LPS-binding test showed that acetylcorynoline does not affect LPS binding on DCs (data not shown), which implies that the suppression of LPS-stimulated DC maturation by acetylcorynoline is not due to the direct interaction between LPS and acetylcorynoline. We also have confirmed that acetylcorynoline has no effects on TLR4 expression on DCs by using western blotting and flow cytometry analysis, (data not shown). The exact mechanisms underlying these results will require further study.

Some reports have indicated that alkaloids, including cepharanthine [36], tetrandrine [37], sinomenine [38], and nicotine [39], have special immunomodulatory effects on DCs. The present study shows that acetylcorynoline is a new member on the list of alkaloids with these effects. Moreover, according our supplement data (Figure S1), acetylcorynoline has been shown to be more effective than other compounds in improving contact hypersensitivity response, and have better potential in pharmaceutics. We also have tested immunosuppressive effect of acetylcorynoline in the LPS-stimulated RAW 264.7 macrophages. At 20 µM acetylcorynoline, LPS-stimulated TNF-α secretion decreased by about 58% (*p*<0.01) (Figure S2). As our data, acetylcorynoline has inhibitory effects on activate macrophages. Data for possible clinical applications of acetylcorynoline is rising. The *in vivo* influences of acetylcorynoline treatment have been showed in a mouse model of experimental liver injury [3]. The report indicates that there is a safety issue in using acetylcorynoline in clinical application. Further clinical trials are required to evaluate the suitability of acetylcorynoline for disease control.

These results support the claims of traditional Chinese medicine practitioners about the utilization of herbs containing acetylcorynoline in the treatment of inflammatory-related diseases [1]. Using this readily obtainable drug supplies a convenient, and low-cost of regulating the immunomodulatory capacity of DCs. In the future, we plan to investigate the precise mechanism by which acetylcorynoline inhibits DC activation and functions in an animal disease model, which may develop the novel immunopharmacological potential of acetylcorynoline for the prevention and treatment of inflammatory and autoimmune disorders.

Recent studies have revealed that incorporation of alkaloids into nanoparticles aids in its oral delivery [32], [33], [35], as these particles can shield the drug from degradation in the digestive tract; as a result of their special absorption mechanism through the lymphatic system, these particles also protect drug from first-pass effect in the liver and allow continued release at the desired site of action. Such techniques will be expected to accelerate improvements in the clinical applications of acetylcorynoline.

## Materials and Methods

### Ethics Statement

This study was carried out in strict accordance with the recommendations in the Guide for the Care and Use of Institutional Animals of China Medical University and the Care and Use of Laboratory Animals of the National Institutes of Health. All animal experiments were conducted according to a protocol approved by the Institutional Animal Care and Use Committee of the China Medical University (Permit Number: 101-32-N).

### Chemicals and antibodies

Synthesized acetylcorynoline (mol. wt. 409.43, 98% purity) was purchased from ChromaDex (Irvine, CA), dissolved in dimethyl sulfoxide (DMSO) to 100 mM, and stored at −20°C as a master stock solution. RPMI 1640-L-gutamine medium, fetal bovine serum, HEPES (pH 7.4), sodium pyruvate, penicillin-streptomycin, nonessential amino acid, and β-mercaptoethanol were purchased from Invitrogen (Grand Island, NY). Recombinant mouse granulocyte-macrophage colony-stimulating factor, recombinant mouse IL-4, and secondary lymphoid-tissue chemokine/chemokine (C-C motif) ligand 21 (SLC/CCL21) were purchased from Prospec (Ness-Ziona, Israel). LPS (from *Escherichia coli* 055:B5), fluorescein isothiocyanate (FITC)-dextran (42 kDa), mitomycin C, 3-(4,5-dimethylthiazol-2-yl)-2,5-diphenyltetrazolium bromide (MTT), 2.4-dinitro-1-fluorobenzene (DNFB), and other chemicals were purchased from Sigma-Aldrich (St. Louis, MO). Phycoerythrin-conjugated antibody to mouse CD11c and FITC-conjugated antibody to mouse MHC class II, CD40, CD86, and isotype-matched control antibodies were purchased from GenWay Biotech (San Diego, CA). The antibodies for IKKα/β, IκBα, JNK, ERK1/2, p38, Akt, and their phosphorylated forms were purchased from Santa Cruz Biotechnology (Santa Cruz, CA). The antibody for mouse β-actin was purchased from Millipore (Billerica, MA).

### Generation of mBM-DCs

C57BL/6 mice (8 weeks old) were maintained in a specific pathogen-free area at the Animal Center of China Medical University (Taichung, Taiwan), and mBM-DCs were acquired as described previously [17], [40].

### Cell viability assay

mBM-DCs were treated with serially diluted acetylcorynoline for 24 h. The final concentration of DMSO in all acetylcorynoline-treated cultures was 0.1% (v/v). Cells were then harvested and stained by using 5 µg/ml propidium iodide or Annexin V kit (Invitrogen, Carlsbad, CA). Cell viability and apoptosis were analyzed by BD LSR II flow cytometry (BD Biosciences, San Jose, CA).

### Cell activation and treatment

Depending on the results of the cell viability assay, mBM-DCs were pretreated with 10 or 20 µM acetylcorynoline for 1 h. The final concentration of DMSO in all acetylcorynoline-treated cultures was 0.1% (v/v). After 1 h of incubation, the cells were washed twice using phosphate-buffered saline, followed by stimulation with 100 ng/ml LPS for the indicated time points. Media and cells were collected for subsequent evaluation of DC activation and analysis of protein expression. Three replicates were included in each experiment.

### Cytokine assay

Acetylcorynoline-pretreated mBM-DCs were stimulated with LPS for 24 h (6 h for TNF-α). The secretion of TNF-α, IL-6, and IL-12p70 in cultured cell media was measured by enzyme-linked immunosorbent assay (ELISA) kits purchased from Invitrogen (Grand Island). The cytokine concentration was evaluated according to the manufacturer's protocol.

### Flow cytometry

Acetylcorynoline-pretreated mBM-DCs were stimulated with LPS for 16 h. The expression of surface molecules on DCs was determined by flow cytometry as described previously [18]. The data were collected for 1×10^4^ cells per sample.

### Endocytosis assay

Acetylcorynoline-pretreated mBM-DCs were stimulated with LPS for 16 h. To analyze the endocytic capacity of DCs, we incubated cells with 1 mg/ml FITC-dextran in fresh medium at 37°C for 1 h. After incubation, cells were washed twice with cold phosphate-buffered saline, stained with phycoerythrin-conjugated anti-CD11c antibody, and analyzed by flow cytometry. Control experiments were performed at 4°C for 1 h. The data were collected for 1×10^4^ cells per sample.

### Allogenic mixed lymphocyte reaction

Splenocytes from the spleens of BALB/c mice (8 weeks old) were isolated by using a mouse T-cell isolation kit (Miltenyi Biotec, Bergisch Gladbach, Germany). Over 90% of cells expressed CD3^+^, as determined by a fluorescence-activated cell sorting system. Acetylcorynoline-pretreated mBM-DCs were stimulated with LPS for 16 h. Cells were then harvested, washed, and incubated with 25 µg/ml mitomycin C for 30 min at 37°C. Finally, the cells were washed and diluted with the prepared splenocytes in a ratio of 1∶50 and 1∶100 in U-bottomed 96-well culture plates for 3 days. Cell proliferation was determined by MTT assay. The absorbance of the dissolved solutions was detected by using a SpectraMax M2 Microplate Reader (Molecular Devices, Silicon Valley, CA) at 570 nm [18].

### Migration assay

Acetylcorynoline-pretreated mBM-DCs were stimulated with LPS for 16 h. For *in vitro* studies of migration [41], [42], DCs in serum-free medium were placed in a 24-well Transwell migration chamber (Corning Costar, Cambridge, MA). RPMI 1640 medium (0.1 ml) containing DCs (1×10^5^ cells) was loaded onto the upper wells. RPMI 1640 medium (0.6 ml) containing SLC/CCL21 (100 ng/ml) and bovine serum albumin (5 mg/ml) was added to the lower wells to induce cell chemotaxis through 5-µm-pore size polycarbonate filters at 37°C. After 4 h, migration was shown as the number of cells that had moved to the lower wells, as counted by flow cytometry.

### NF-κB assay

Acetylcorynoline-pretreated mBM-DCs were stimulated with LPS for 1 h. The nuclear protein of cells was extracted by using the Nuclear Extraction Kit (Affymetrix-Panomics, Santa Clara, CA). NF-κB p65 binding activity was determined with the Universal EZ-TFA Transcription Factor Assay Colorimetric kit according to the manufacturer's instructions (Millipore, Billerica, MA) and quantified by absorbance (450 nm) with a microplate reader.

### Western blot analysis

Acetylcorynoline-pretreated mBM-DCs were stimulated with LPS for 1 h and then lysed in radioimmunoprecipitation assay buffer (Millipore) to extract proteins. The concentration of whole cell lysates was calculated by using the RC DC Protein Assay Kit (Bio-Rad Life Science, Hercules, CA). Thirty micrograms of protein per sample was loaded onto an SDS-PAGE gel and analyzed by Western blotting for IKKα/β, IκBα, JNK, ERK1/2, p38, Akt, and their phosphorylated forms as previously described [18]. Signals were assessed by using a UVP BioSpectrum Imaging System (Upland, CA).

### Inhibitor treatment

IKK-2 Inhibitor IV and p38 MAPK inhibitor SB203580 were purchased from Santa Cruz Biotechnology (Santa Cruz, CA) and were dissolved in DMSO. Inhibitors were titrated at concentrations ranging from 0.1 to 40 mM. A dose of 2 mM for IKK-2 Inhibitor IV or 20 mM for SB203580 was used for experiments as this was the lowest concentration that could completely block kinase activity. For inhibitor analysis, mBM-DCs were preincubated with the indicated inhibitors for 30 min, and then incubated with or without 20 µM acetylcorynoline. After 1 h of incubation, the cells followed by stimulation with 100 ng/ml LPS for 16 h. Inhibitors were removed by twice washing mBM-DCs with 2% FBS/PBS. Cells were collected and performed allogenic mixed lymphocyte reaction.

### Contact hypersensitivity assay

Contact hypersensitivity (CHS) test induced by DNFB was described previously [19]. The shaved belly of mice were painted with 20 µl of vehicle (acetone/olive oil = 4/1), 0.5% (w/v) DNFB, 0.5% DNFB plus 0.1% (v/v) DMSO, or 0.5% DNFB plus acetylcorynoline (100 µg) for sensitization. After 5 day, all mice were challenged by painting on the backs of shaved ears with 10 µl 0.2% DNFB (∼1 cm diameter). Mice that were not sensitized but were challenged with DNFB provided as negative controls. CHS response was measured 24 h later by hematoxylin and eosin staining. Swelling of the ear was calculated by subtracting the thickness before challenge.

### Statistical analysis

All statistical analyses are expressed as mean ± standard deviation (SD) from three independent tests. Three replicates were done of each test. The differences between two means were determined by student's *t*-test. Values of *p*<0.05 were determined to be statistically significant.

## Supporting Information

Figure S1 **Inhibitory effect of acetylcorynoline, cepharanthine, tetrandrine, sinomenine, or nicotine on the contact hypersensitivity response in mice.** Contact hypersensitivity response was showed by thickness of the challenged ear was calculated. Mice that were not sensitized but were challenged with DNFB provided as negative controls. The data represent the mean ± SD (n = 3). A hash (#) indicates significant differences between unsensitized and sensitized mice (*p*<0.01); an asterisk (*) indicates significant differences between the DNFB-challenged control samples and DNFB plus acetylcorynoline, cepharanthine, tetrandrine, or nicotine-challenged samples (^*^ *p*<0.05); an caret (∧) indicates significant differences between DNFB plus acetylcorynoline samples and DNFB plus cepharanthine, tetrandrine, sinomenine, or nicotine-challenged samples (^**^ *p*<0.05).
(DOC)

Figure S2 **Inhibitory effects of acetylcorynoline on TNF-α secretion in LPS-stimulated RAW 264.7 cells.** RAW 264.7 cells (Mouse leukaemic monocyte macrophage cell line) were purchased from the Bioresources Collection and Research Center (BCRC, Hsin Chu, Taiwan). Cells were maintained using DMEM, supplemented with 2 mM L-glutamine, 10% fetal bovine serum and 1% penicillin–streptomycin at 37°C, 5% CO2. A MTT assay was used to evaluate the cytotoxicity of acetylcorynoline. Cell viability was not significantly changed by 24-h treatment with up to 20 µM acetylcorynoline (data not shown). RAW 264.7 cells were pretreated with 10 or 20 µM acetylcorynoline. After 1 h of incubation, the cells were washed, followed by stimulation with 1 µg/ml LPS for 20 h. Media were collected and assayed for TNF-α levels by using as ELISA kit. The data represent the mean ± SD (n = 3). A hash (#) indicates significant differences between LPS-stimulated and unstimulated cells (*p*<0.001); an asterisk (*) indicates significant differences between the LPS-stimulated control samples and acetylcorynoline-pretreated, LPS-stimulated samples (^*^ *p*<0.05, ^**^ *p*<0.01).
(DOC)
