# Supplementary material for: Acetylcorynoline Impairs the Maturation of Mouse Bone Marrow-Derived Dendritic Cells via Suppression of IκB Kinase and Mitogen-Activated Protein Kinase Activities
Source: PLoS One. 2013 Mar 5;8(3):e58398. doi: 10.1371/journal.pone.0058398 (PMC3589392; doi:10.1371/journal.pone.0058398)
Supplement: Figure S1 — Inhibitory effect of acetylcorynoline, cepharanthine, tetrandrine, sinomenine, or nicotine on the contact hypersensitivity response in mice. Contact hypersensitivity response was showed by thickness of the challenged ear was calculated. Mice that were not sensitized but were challenged with DNFB provided as negative controls. The data represent the mean ± SD (n = 3). A hash (#) indicates significant differences between unsensitized and sensitized mice (p<0.01); an asterisk (*) indicates significant differences between the DNFB-challenged control samples and DNFB plus acetylcorynoline, cepharanthine, tetrandrine, or nicotine-challenged samples (* p<0.05); an caret (∧) indicates significant differences between DNFB plus acetylcorynoline samples and DNFB plus cepharanthine, tetrandrine, sinomenine, or nicotine-challenged samples (** p<0.05). (DOC) [file pone.0058398.s001.doc]

**
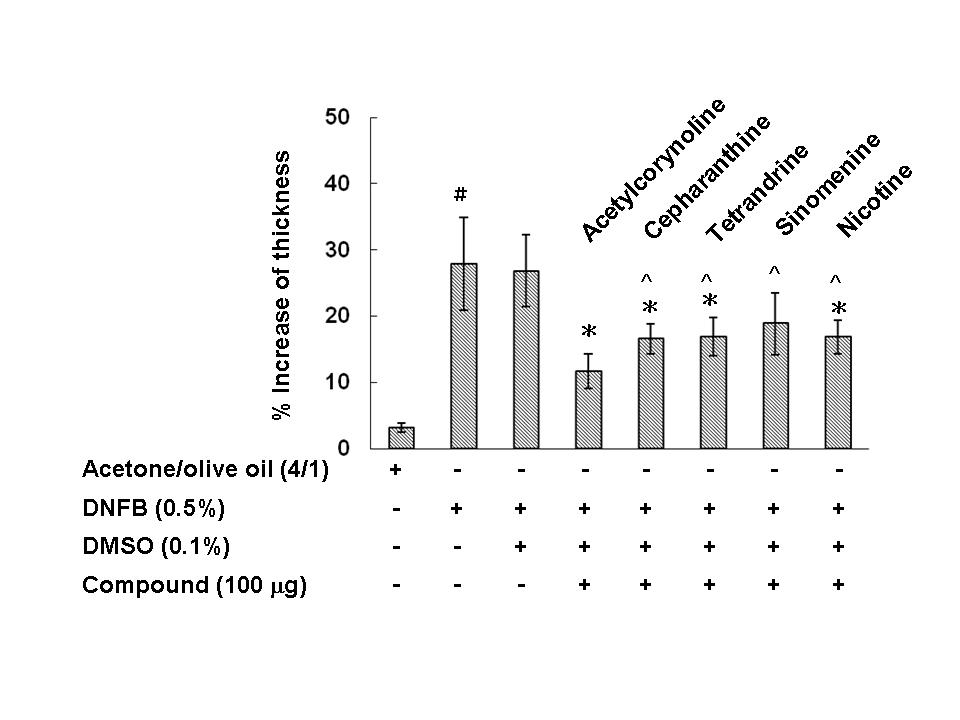
**

**Figure S1. Inhibitory effect of acetylcorynoline, cepharanthine, tetrandrine, sinomenine, or nicotine on the contact hypersensitivity response in mice.** Contact hypersensitivity response was showed by thickness of the challenged ear was calculated. Mice that were not sensitized but were challenged with DNFB provided as negative controls. The data represent the mean ± SD (n = 3). A hash (#) indicates significant differences between unsensitized and sensitized mice (*p* < 0.01); an asterisk (*) indicates significant differences between the DNFB-challenged control samples and DNFB plus acetylcorynoline, cepharanthine, tetrandrine, or nicotine-challenged samples (^*^*p* < 0.05); an caret (∧) indicates significant differences between DNFB plus acetylcorynoline samples and DNFB plus cepharanthine, tetrandrine, sinomenine, or nicotine-challenged samples (^**^*p* < 0.05).
