# Supplementary material for: Acetylcorynoline Impairs the Maturation of Mouse Bone Marrow-Derived Dendritic Cells via Suppression of IκB Kinase and Mitogen-Activated Protein Kinase Activities
Source: PLoS One. 2013 Mar 5;8(3):e58398. doi: 10.1371/journal.pone.0058398 (PMC3589392; doi:10.1371/journal.pone.0058398)
Supplement: Figure S2 — Inhibitory effects of acetylcorynoline on TNF-α secretion in LPS-stimulated RAW 264.7 cells. RAW 264.7 cells (Mouse leukaemic monocyte macrophage cell line) were purchased from the Bioresources Collection and Research Center (BCRC, Hsin Chu, Taiwan). Cells were maintained using DMEM, supplemented with 2 mM L-glutamine, 10% fetal bovine serum and 1% penicillin–streptomycin at 37°C, 5% CO2. A MTT assay was used to evaluate the cytotoxicity of acetylcorynoline. Cell viability was not significantly changed by 24-h treatment with up to 20 µM acetylcorynoline (data not shown). RAW 264.7 cells were pretreated with 10 or 20 µM acetylcorynoline. After 1 h of incubation, the cells were washed, followed by stimulation with 1 µg/ml LPS for 20 h. Media were collected and assayed for TNF-α levels by using as ELISA kit. The data represent the mean ± SD (n = 3). A hash (#) indicates significant differences between LPS-stimulated and unstimulated cells (p<0.001); an asterisk (*) indicates significant differences between the LPS-stimulated control samples and acetylcorynoline-pretreated, LPS-stimulated samples (* p<0.05, ** p<0.01). (DOC) [file pone.0058398.s002.doc]

**
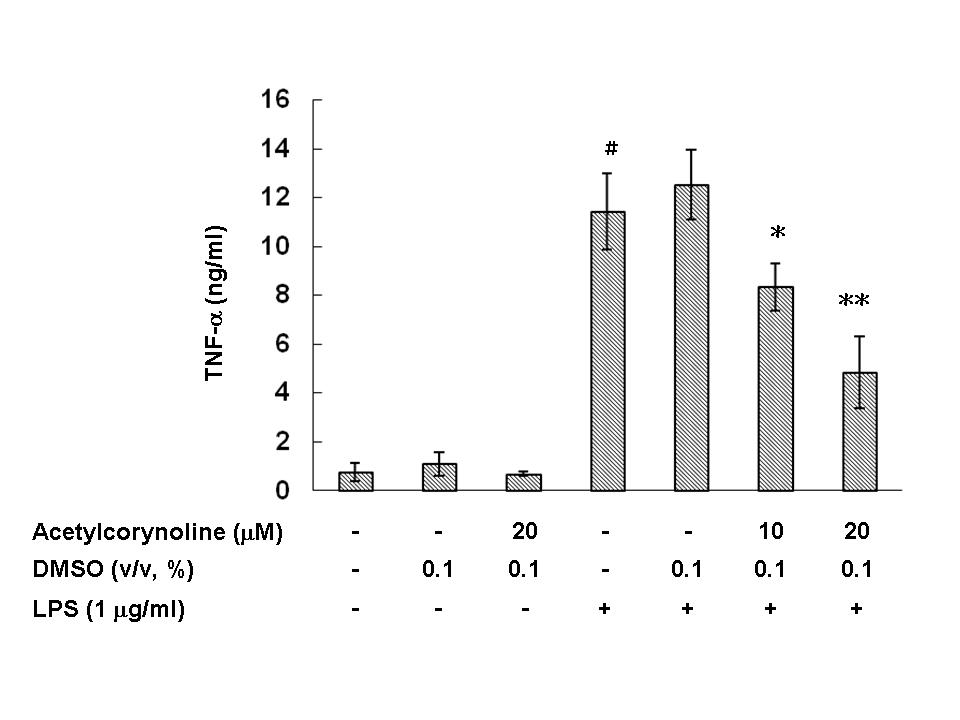
**

**Figure S2. Inhibitory effects of acetylcorynoline on TNF-α secretion in LPS-stimulated RAW 264.7 cells.** RAW 264.7 cells (Mouse leukaemic monocyte macrophage cell line) were purchased from the Bioresources Collection and Research Center (BCRC, Hsin Chu, Taiwan). Cells were maintained using DMEM, supplemented with 2 mM L-glutamine, 10% fetal bovine serum and 1% penicillin–streptomycin at 37 °C, 5% CO2. A MTT assay was used to evaluate the cytotoxicity of acetylcorynoline. Cell viability was not significantly changed by 24-h treatment with up to 20 μM acetylcorynoline (data not shown). RAW 264.7 cells were pretreated with 10 or 20 μM acetylcorynoline. After 1 h of incubation, the cells were washed, followed by stimulation with 1 μg/ml LPS for 20 h. Media were collected and assayed for TNF-α levels by using as ELISA kit. The data represent the mean ± SD (n = 3). A hash (#) indicates significant differences between LPS-stimulated and unstimulated cells (*p* < 0.001); an asterisk (*) indicates significant differences between the LPS-stimulated control samples and acetylcorynoline-pretreated, LPS-stimulated samples (^*^*p* < 0.05, ^**^*p* < 0.01).
